# Therapeutic modulation of the gut microbiota by traditional Chinese medicine in the management of cholestatic liver injury

**DOI:** 10.3389/fcimb.2026.1807162

**Published:** 2026-04-15

**Authors:** Xiyan Ding, Jiaming Wang, Yicui Wang, Huaming Xu, Yanxin Liu

**Affiliations:** School of Medicine, Henan University of Chinese Medicine, Zhengzhou, Henan, China

**Keywords:** advances, cholestatic liver disease, gut microbiota, gut-liver axis, traditional Chinese medicine

## Abstract

Cholestatic liver injury (CLI) is a complex pathology characterized by impaired bile excretion and a lack of effective curative therapies. Emerging evidence indicates that the gut microbiota plays a critical role in the pathogenesis of CLI via the gut-liver axis. Specifically, gut dysbiosis disrupts bile acid homeostasis, triggers immune-mediated inflammation, exacerbates oxidative stress, and dysregulates multiple signaling pathways, thereby accelerating hepatic damage. Traditional Chinese Medicine (TCM) offers a distinct therapeutic advantage through its multi-component, multi-target mechanisms. Many studies have shown that TCM herbal extracts and some formulas can attenuate CLI by restructuring the gut microbiome. These interventions work by promoting beneficial bacterial proliferation, restoring intestinal barrier integrity, modulating bile acid receptors, and suppressing inflammation and fibrosis. This review synthesizes current mechanisms linking gut dysbiosis to CLI and evaluates recent advances in TCM-based strategies that target the gut microbiota, offering theoretical insights for novel clinical interventions.

## 1 Introduction

CLI is a prevalent hepatobiliary pathology characterized by impaired intrahepatic or extrahepatic bile flow. This obstruction leads to the hepatic and systemic accumulation of cytotoxic substances, particularly hydrophobic bile acids (Wu et al., 2024), which subsequently precipitate hepatocyte necrosis and inflammation. Chronic, unresolved cholestasis can progress to fibrosis, cirrhosis, and ultimately, liver failure (Verkade et al., 2023; Trauner and Fuchs, 2022). Consequently, elucidating the pathogenesis of CLI and identifying novel therapeutic targets is of critical importance in hepatology. Recently, the gut-liver axis has emerged as a crucial framework for understanding hepatic pathophysiology (Benedé-Ubieto et al., 2024). As the liver is the sole organ receiving direct blood flow from the intestines via the portal vein, it maintains a robust anatomical and functional reciprocity with the gut. Dysbiosis of the gut microbiota is not merely a consequence of CLI but an active contributor of disease progression (Shen et al., 2024). Alterations in gut microbiome composition exacerbate liver injury by impairing bile acid homeostasis, inducing immune-mediated inflammatory cascades, and amplifying oxidative stress (Leung et al., 2023). Thus, targeted modulation of the gut microbiota represents a promising therapeutic strategy for CLI.

TCM has been utilized extensively in the clinical management of liver disease. The “holistic” approach of TCM aligns closely with the modern gut-liver axis paradigm (Zhou et al., 2025). TCM interventions—ranging from herbal extracts to Chinese herbal compound formula—exert pleiotropic effects via multi-component and multi-target mechanisms. Crucially, oral TCM formulations interact directly with the gut microbiota prior to systemic absorption, effectively restructuring microbial communities and restoring intestinal barrier integrity to improve liver function. This review synthesizes current knowledge regarding the pathogenic role of gut microbiota in CLI and evaluates recent advances in TCM-based strategies that attenuate liver injury through microbiome regulation, providing a theoretical foundation for novel clinical interventions.

## 2 Cholestatic liver injury and gut microbiota

### 2.1 Cholestatic liver injury

CLI represents a spectrum of hepatobiliary diseases characterized primarily by impaired bile flow dynamics (Zhang et al., 2025a). The pathophysiological essence of CLI lies in the compromised bile secretory function of hepatocytes or excretory dysfunction of the biliary system, leading to the pathological accumulation of hydrophobic bile acids and other cytotoxic metabolites in the hepatic parenchyma and systemic circulation. This accumulation subsequently induces mitochondrial dysfunction, hepatocyte necrosis, and a sustained inflammatory cascade (Tilg et al., 2022; Collins et al., 2023). Diseases in this category often present insidiously, with initial manifestations limited to biochemical abnormalities such as elevated alkaline phosphatase (ALP) and Gamma-Glutamyl Transferase (GGT); as the condition progresses, patients may develop intractable pruritus, chronic fatigue, and hyperbilirubinemia, which significantly compromise their quality of life (Gulamhusein and Hirschfield, 2020). Without effective intervention, persistent cholestasis drives hepatic stellate cell activation, leading to progressive liver fibrosis, cirrhosis, and ultimately liver failure (Akkız et al., 2024). This disease spectrum encompasses various subtypes, primarily including primary biliary cholangitis, primary sclerosing cholangitis, and hereditary cholestasis (Alam and Lal, 2022; Park et al., 2022). Among adult patient populations, primary biliary cholangitis and primary sclerosing cholangitis are the important though infrequent. The former exhibits significant sexual dimorphism, occurring predominantly in women, whereas the latter is frequently associated with a high rate of comorbidity with inflammatory bowel disease (Trivedi et al., 2016). Current therapeutic options remain relatively limited. The first-line agent, ursodeoxycholic acid, yields an inadequate initial biochemical response in approximately 40% of patients (Roberts et al., 2024). Moreover, the incidence of CLI has shown a gradual increasing trend and has become an established indication for liver transplantation in many Western countries (Mol et al., 2025).

### 2.2 Gut microbiota

The gut microbiota constitutes a microecosystem that resides stably within the human digestive tract. It is predominantly composed of commensal bacteria and also encompasses viruses, archaea, fungi, and other single−celled microorganisms (Khalil et al., 2024). The microbial community and the host are deeply involved in processes such as digestion and absorption, inflammation and immune regulation, and metabolic transformation, playing an indispensable role in maintaining human physiological homeostasis (Yang and Kweon, 2016; Paul et al., 2025). Notably, this microecosystem exhibits distinct age−related characteristics throughout the host’s lifespan, with its community structure and functional diversity demonstrating significant dynamic successional patterns during infancy, adulthood, and old age (Bradley and Haran, 2024). Despite such dynamic changes, in healthy adults, the gut microbial community generally exhibits a stable core composition, predominantly comprising the phyla *Firmicutes* and *Bacteroidetes*, along with minor proportions of *Proteobacteria*, *Verrucomicrobia*, *Actinobacteria*, and other taxa (Fusco et al., 2023). As dominant microbial phyla, the *Firmicutes* and *Bacteroidetes* not only prevail in abundance but also exhibit remarkable functional specificity and complementarity in degrading dietary fibers, producing bioactive metabolites, and maintaining intestinal ecological stability (Jensen et al., 2024). The *Firmicutes* phylum primarily consists of Gram-positive bacteria, with representative members including *Clostridium*, and *Faecalibacterium prausnitzii* (Martin-Gallausiaux et al., 2021). These microorganisms are mainly responsible for converting dietary fibers into short-chain fatty acids (SCFAs) such as butyrate, thereby providing a critical energy source for intestinal epithelial cells and modulating the host’s inflammatory responses (Stoeva et al., 2021). In contrast, the phylum *Bacteroidetes* is predominantly composed of *Gram-negative bacteria*, including genera such as *Bacteroides*. The genomes of this phylum are enriched with polysaccharide utilization loci (PULs), which enable these bacteria to degrade host glycans and complex plant polysaccharides, producing acetate and propionate as their primary metabolic end-products (Zafar and Saier, 2021). Given their central roles in the gut microbiota, the relative ratio of *Firmicutes* to *Bacteroidetes* (F/B ratio) has been widely adopted as a key biomarker for assessing gut microbial balance and predicting the risk of obesity and metabolic syndrome (McCallum and Tropini, 2024; Crovesy et al., 2020).

### 2.3 Gut-liver axis

The gut-liver axis refers to a closely integrated system formed by the anatomical structures and functional metabolism of the gut and the liver (Hsu and Schnabl, 2023). The two organs maintain bidirectional connection through the biliary system, portal venous circulation, and systemic circulation (Albillos et al., 2020). The liver secretes bile acids and bioactive molecules it synthesizes into the intestine via the biliary system, thereby directly modulating the intestinal environment (Larabi et al., 2023). Nutrients absorbed by the gut, microbial metabolites, and exogenous antigens are transported directly to the liver through the portal venous system (Kandalgaonkar et al., 2024). Given that approximately 70% of the liver’s blood supply originates from the intestines, this unique hemodynamic feature positions the liver as the first line of defense against gut-derived substances and establishes the indispensable role of gut microbiota in regulating hepatic function (Schnabl, 2013). Under physiological conditions, the gut-liver axis maintains a highly coordinated and dynamic equilibrium (Zhu et al., 2023). Bile acids secreted by the liver not only serve as key mediators of lipid digestion but also act as important signaling molecules that reshape the gut microbiota by inhibiting the growth of pathogenic bacteria and maintaining mucosal barrier integrity (Grüner and Mattner, 2021). Conversely, the gut microbiota, often regarded as a virtual metabolic organ, participates in the biotransformation and reabsorption of bile acids through the enterohepatic circulation. It also delivers beneficial metabolites such as SCFAs back to the liver, thereby modulating hepatic energy metabolism and immune tolerance (Wang et al., 2025a, Wang et al., 2022a). This interplay between the gut microbiota and bile acids plays a crucial role in maintaining systemic homeostasis. Dysbiosis can severely disrupt the enterohepatic circulation and metabolism of bile acids. When intestinal barrier function is compromised, it leads to endotoxin translocation, subsequently activating hepatic immune responses and triggering inflammatory cascades and oxidative stress injury (Yang et al., 2025; Liu et al., 2024). This pathological assault originating from the gut constitutes a core mechanism driving the onset and progression of CLI.

## 3 Mechanisms of gut microbiota modulation in CLI

### 3.1 Bile acid metabolism

**Figure 1:**
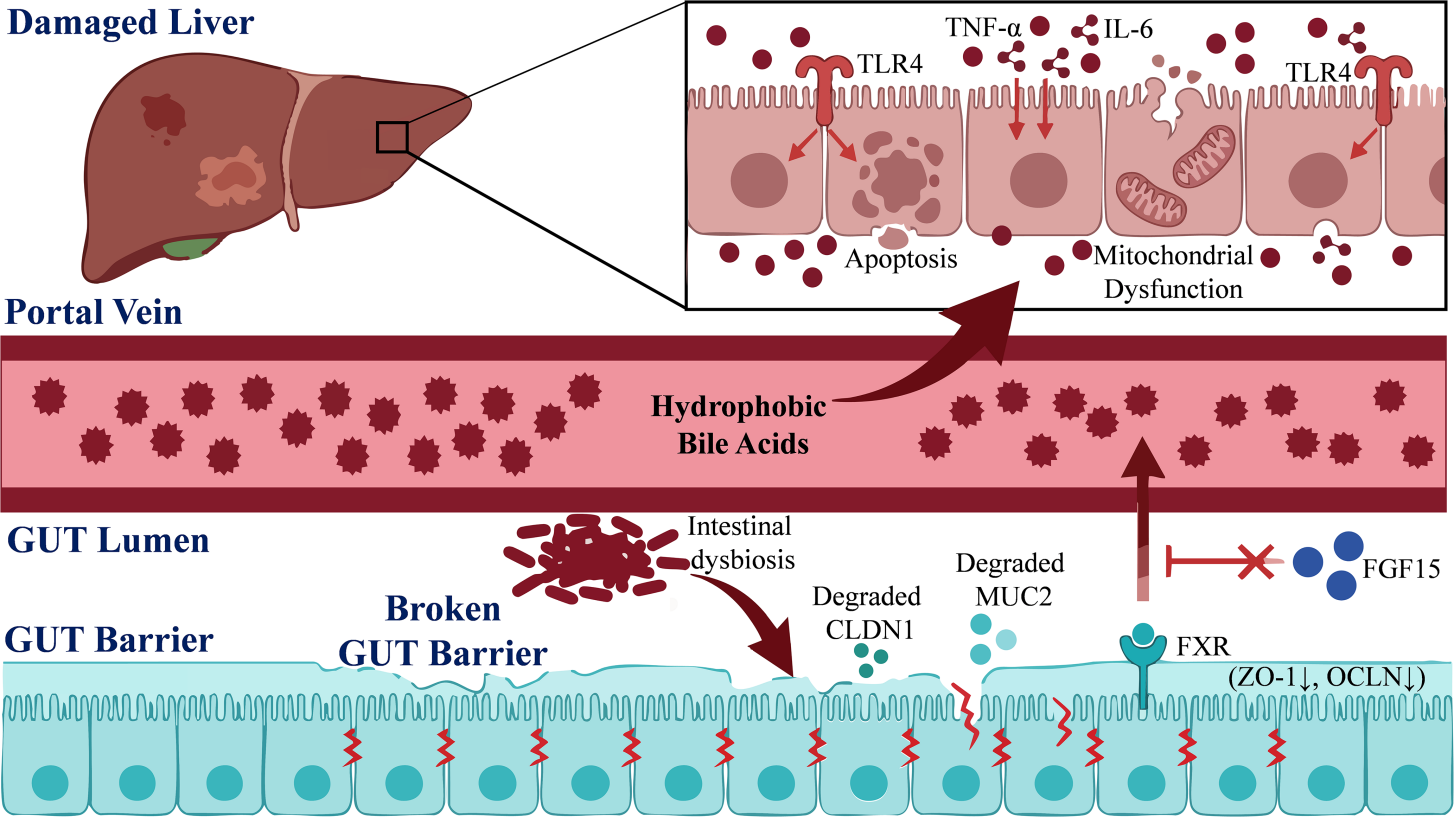
The gut microbiota influences CLI through the modulation of bile acids.

The metabolic processing of primary bile acids synthesized by the liver after they enter the intestine is highly dependent on the functional activity of the gut microbiota (Nie et al., 2024). In particular, microbial enzymes such as bile salt hydrolase and 7α-dehydroxylase are responsible for converting primary bile acids into secondary bile acids, including deoxycholic acid (DCA) and lithocholic acid (LCA) (Jiao et al., 2018). When highly hydrophobic secondary bile acids accumulate abnormally in hepatocytes, they can disrupt hepatocyte membrane and mitochondrial function (Lu et al., 2025), induce hepatic inflammation (Zeng et al., 2020), and lead to hepatocyte necrosis and apoptosis (Woolbright and Jaeschke, 2019). In the context of CLI, the gut microbiota structure undergoes significant dysbiosis, characterized by a decreased abundance of beneficial microbial communities and an increased abundance of potentially pathogenic bacteria. This imbalance is positively correlated with serum total bile acid (TBA) levels, demonstrating an association between gut dysbiosis and disordered bile acid metabolism (Li et al., 2023). Such dysbiosis further impairs intestinal barrier function and significantly alters bile acid metabolism. This dysbiosis reduces the capability of gut microbes to convert primary bile acids into secondary bile acids (Chen et al., 2020), resulting in a decreased ratio of secondary bile acids. Consequently, hepatic bile acid homeostasis is disrupted, leading to relative accumulation of hydrophobic bile acids, which exacerbates liver inflammation and injury (Yang et al., 2024). These toxic bile acids directly exacerbate hepatocyte injury and death by activating inflammatory signaling pathways, notably through upregulation of tumor necrosis factor-α (TNF-α), interleukin-6 (IL-6), and Toll-like receptor 4 (TLR4) expression (Ali et al., 2021). Furthermore, bile acids act as signaling molecules that can negatively regulate their own synthesis and secretion through the activation of receptors such as the Farnesoid X Receptor (FXR). Notably, LCA and DCA, which are metabolites derived from gut microbiota, serve as natural ligands for FXR (Fan et al., 2021). Gut dysbiosis may disrupt this feedback mechanism. Under cholestatic conditions induced by glycochenodeoxycholate, an increase in pathogenic bacteria impairs intestinal barrier integrity, as evidenced by reduced expression of Mucin 2 (MUC2), Claudin-1 (CLDN1), Occludin (OCLN), and Zonula Occludens-1 (ZO-1). This impairment directly suppresses the expression of FXR and its downstream target, Fibroblast Growth Factor 15 (FGF15), thereby damaging the intestinal FXR signaling pathway and exacerbating systemic bile acid metabolic dysregulation (Xie et al., 2025). Therefore, the gut microbiota plays a central role in CLI by modulating bile acid metabolism (**Figure 1**).

### 3.2 Immune and inflammatory mechanisms

**Figure 2:**
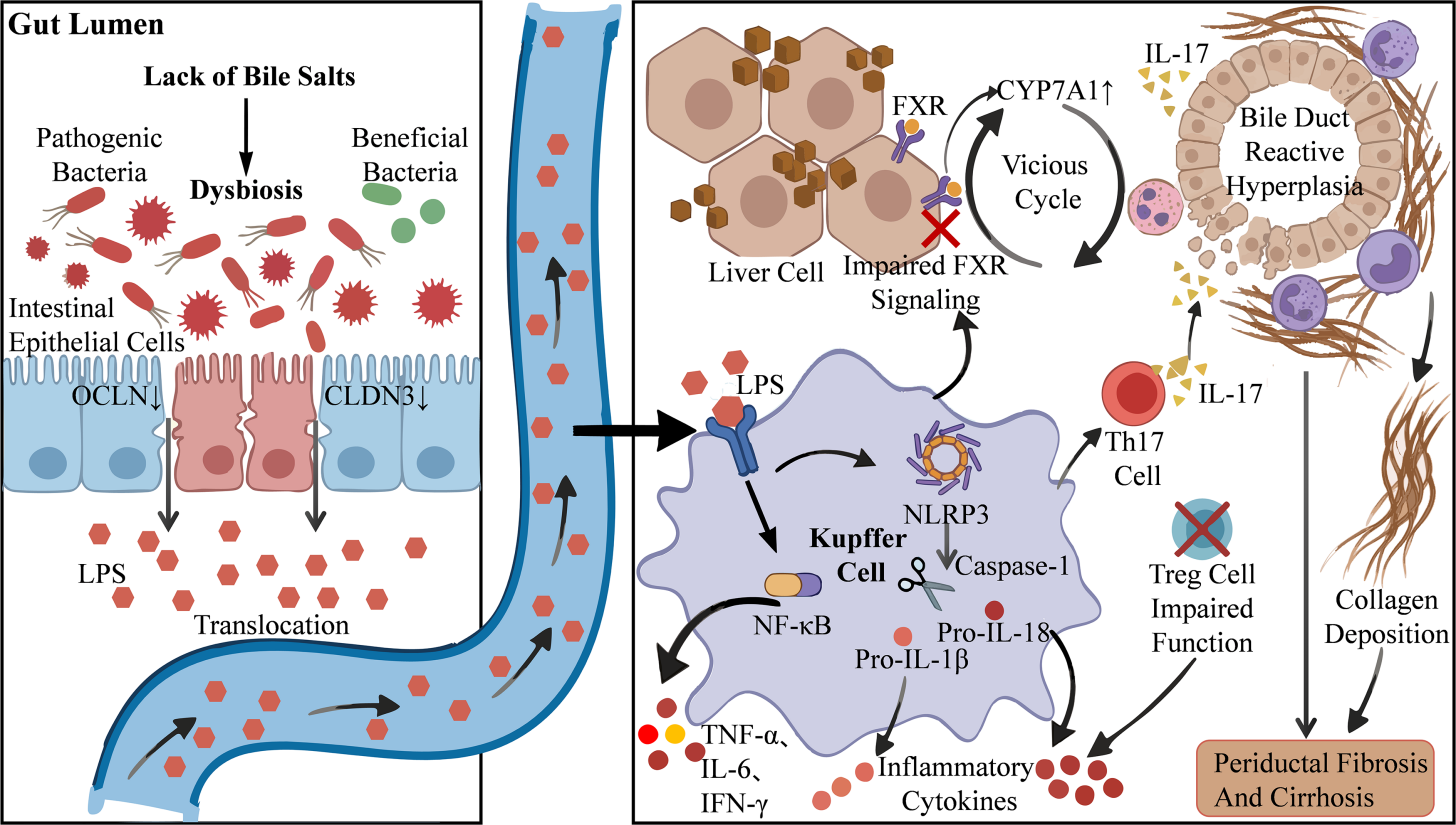
The gut microbiota influences CLI through inflammatory and immune pathways.

Within the interplay of gut microbiota and CLI, immune and inflammatory responses form a sophisticated and intricate regulatory network via the gut-liver axis (Cui et al., 2025a). Under cholestasis, the diminished bacteriostatic effect of bile acids in the gut leads to excessive proliferation of pathogenic bacteria and a marked reduction in beneficial flora. This shift in microbial structure impairs the metabolic functions of the microbiota and compromises intestinal barrier integrity (Zou et al., 2025). Downregulation of proteins such as OCLN and claudin-3 increases paracellular permeability of the intestinal epithelium (Hahn et al., 2020). Consequently, bacterial products like lipopolysaccharides (LPS) translocate into the portal vein system and activate hepatic Kupffer cells via TLR4. This triggers the intracellular nuclear factor kappa-B (NF-κB) signaling pathway, leading to a substantial release of pro-inflammatory cytokines such as TNF-α, IL-6, and interferon-gamma (Chang et al., 2025). This inflammatory microenvironment further promotes the assembly of the NOD-like receptor thermal protein domain associated protein 3 (NLRP3) inflammasome. Upon activation of caspase-1, it cleaves the precursors pro-interleukin-1β and pro-interleukin-18 to generate bioactive inflammatory cytokines, thereby amplifying the inflammatory cascade (Isaacs-Ten et al., 2020). In the immune response phase, the differentiation of T helper 17 (Th17) cells is markedly enhanced. The interleukin-17 (IL-17) secreted by these cells acts directly on cholangiocytes, inducing apoptosis and promoting the infiltration of neutrophils and monocytes/macrophages around the bile ducts (He et al., 2025). Concurrently, the function of regulatory T cells (Tregs) is impaired, accompanied by an imbalance in the ratio of CD4+ and CD25+ T cells. This undermines immune tolerance mechanisms and leads to dysregulated inflammatory responses (Huang et al., 2021; Tan et al., 2025) (**Figure 2**).

Furthermore, a vicious cycle develops between bile acid metabolism dysregulation and immune dysfunction (Zheng et al., 2024). The accumulation of hydrophobic bile acids in the liver activates key receptors such as FXR, G protein-coupled bile acid receptor 1, and Takeda G protein-coupled receptor 5 (TGR5), which subsequently regulate the expression of fibrosis-related genes (Juanola et al., 2021). Impairment of the FXR signaling pathway further upregulates the expression of cytochrome P450 7A1 (CYP7A1), leading to enhanced bile acid synthesis and establishing a positive feedback loop that amplifies hepatic injury (Wang et al., 2025b). In the cholestatic microenvironment, activation of the macrophage-inflammasome axis not only directly induces intrahepatic inflammatory responses but also reshapes gut microbiota structure by modulating intestinal immune responses, thereby altering the efficiency of the enterohepatic circulation of bile acids (Bozward et al., 2021). This sustained hepatic-intestinal immune dysregulation ultimately leads to reactive bile duct hyperplasia and excessive collagen deposition, driving disease progression toward periductal fibrosis and cirrhosis (Guo et al., 2024). In summary, immune inflammation serves as a central driver that intricately links gut dysbiosis with hepatobiliary injury, providing a solid theoretical foundation for targeting gut microbiota in the treatment of CLI (**Figure 2**).

### 3.3 Oxidative stress

**Figure 3:**
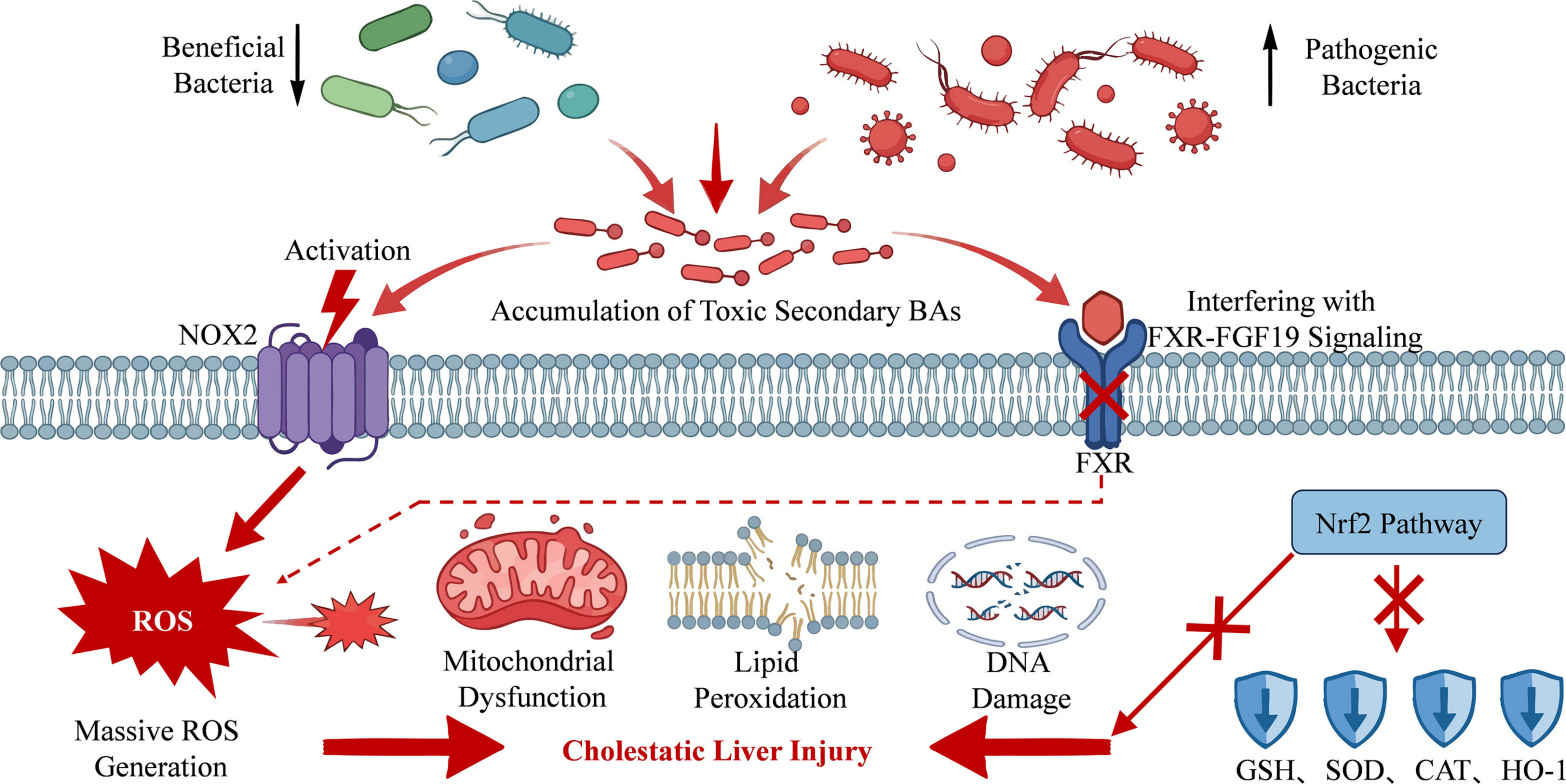
The gut microbiota influences CLI through oxidative stress.

Oxidative stress is defined as an imbalance between the production of reactive oxygen species (ROS) and the capacity of the antioxidant defense system to neutralize them (Garcia-Llorens et al., 2025). This disruption of the oxidative-antioxidative balance leads to the excessive accumulation of free radicals, which subsequently inflicts damage on critical biomolecules such as lipids, proteins, and DNA within cells (Feng et al., 2025). Under cholestatic conditions, toxic bile acids such as DCA or chenodeoxycholic acid (CDCA), which accumulate in the liver, promote the substantial generation of ROS like superoxide and hydrogen peroxide (H_2_O_2_) by activating NADPH oxidases, including NADPH oxidase 2 (NOX2). This leads to a sharp increase in oxidative stress (Li et al., 2024). Such oxidative stress not only directly triggers lipid peroxidation, DNA damage, and mitochondrial dysfunction in hepatocytes but also further disrupts cell membrane integrity and activates apoptotic signaling pathways, thereby exacerbating liver injury (Tokaç et al., 2015). Intestinal dysbiosis, characterized by a reduction in beneficial bacteria and the overgrowth of harmful bacteria, alters the bile acid metabolic profile, leading to an increased proportion of toxic secondary bile acids. These bile acids indirectly induce ROS production by interfering with the signaling of the FXR-fibroblast growth factor 19 (FGF19) axis (Hernandez et al., 2020; Tang et al., 2025). Such persistent oxidative stress further impairs the endogenous antioxidant defense system in hepatocytes, diminishing the activities of superoxide dismutase (SOD), catalase (CAT), and glutathione (GSH), while suppressing the expression of phase II detoxifying enzymes such as heme oxygenase-1 (HO-1), ultimately exacerbating hepatocyte injury (Đurašević et al., 2021; Wu et al., 2025). Additionally, gut microbiota-derived metabolites can enhance antioxidant capacity by upregulating the nuclear factor erythroid-2-related factor 2 (Nrf2) pathway (Saeedi et al., 2020; Kim et al., 2020). In summary, oxidative stress-mediated disruption of the “gut microbiota–bile acid” metabolic axis represents a key mechanism in the pathogenesis of CLI. Targeting this pathway may therefore serve as a promising therapeutic strategy in the future (**Figure 3**).

### 3.4 Other mechanisms

Recent studies have gradually uncovered other important molecular signaling pathways, genetic factors, and gut microbiota interactions in CLI. Among these, the gut microbiota itself has been shown to possess the pathogenic potential to directly drive liver injury (Luo and You, 2023). Gut microbiota derived from CLI patients can independently induce liver injury, often accompanied by enrichment of immune pathways such as Toll-like receptor signaling, highlighting the direct role of the microbiota in driving immune dysregulation (Jiang et al., 2024). Furthermore, host-derived microRNAs (miRNAs) have been found to influence the progression of liver injury by modulating the gut microbiota (Kennedy et al., 2016). Specifically, miRNA-21 can directly suppress the growth of *Lactobacillus* in the small intestine. Conversely, miRNA-21 knockout mice exhibit attenuated bile duct ligation-induced cholestatic liver fibrosis, which is associated with an increased abundance of *Lactobacillus*. This finding confirms that host miRNAs can intervene in the pathological process of CLI by remodeling the composition of the gut microbiota (Santos et al., 2020). In addition, host miRNA-21 impairs the intestinal barrier and exacerbates microbial dysbiosis. Therefore, inhibiting endogenous miRNA-21 represents a potential therapeutic strategy for CLI (Casado-Bedmar et al., 2024).

The sphingosine-1-phosphate receptor 2 (S1PR2) is aberrantly activated under cholestatic conditions and serves as a critical mediator in amplifying inflammatory responses and fibrotic injury through the gut-liver axis (Yang et al., 2023b). Pathologically accumulated conjugated bile acids in the liver can specifically activate S1PR2, which in turn drives the activation of hepatic stellate cells via the p38 mitogen-activated protein kinase/Yes-associated protein signaling axis, ultimately promoting the development and progression of liver fibrosis (Cao et al., 2024; Yang et al., 2023a).

Furthermore, the Small Heterodimer Partner (SHP) plays a key regulatory role in maintaining bile acid metabolic homeostasis and providing hepatoprotection (Nguyen et al., 2021). Although SHP deficiency does not directly induce liver injury, its functional loss significantly exacerbates enterohepatic histopathological damage caused by exogenous factors through the disruption of serum metabolic profiles and the balance of intestinal flora, thereby revealing an indispensable and non-redundant protective function of SHP in maintaining the homeostasis of the gut-liver axis (Wei et al., 2024).

In addition to specific molecular regulators, the host’s genetic background has also been demonstrated to profoundly participate in the progression of liver diseases by reshaping the intestinal microenvironment (Zhang et al., 2023). In carriers of high-risk alleles, the abundance of the genus *Veillonella* is positively correlated with the severity of cirrhosis, providing compelling evidence for the “genetic background-microbiota-disease progression” axis (Huang et al., 2022). These mechanisms are intricately interconnected, collectively constituting the multi−layered network of CLI and offering novel perspectives for targeted therapies.

## 4 Progress in TCM ameliorating CLI via modulation of gut microbiota

TCM (**Table 1**) exerts a prominent hepatoprotective effect against CLI by virtue of its diverse active components and formulations, all of which act through the core mechanism of modulating the gut microbiota structure and its metabolic functions. These TCM-based interventions remodel the gut microbial community, repair intestinal barrier integrity, and regulate bile acid metabolism and related signaling pathways, thereby multi-targetedly alleviating hepatic inflammation, fibrosis and cholestatic damage. The following sections detail the specific mechanisms and efficacy of representative TCM interventions, organized by source and formulation.

**Table 1:** Mechanisms and primary effects of TCM-based therapies on gut microbiota in cholestatic liver injury.

Category	Name	Target	Mechanism of action	Primary effects	Reference
Single herbal extracts	Gardenia jasminoides Ellis Polysaccharide	*Bacteroidetes, Lachnospiraceae ↑;* *Proteobacteria, Enterobacteriaceae, Enterococcaceae ↓*	ZO-1, OCLN ↑; TLR4/NF-κB pathway ↓; FXR, PXR and downstream Bsep, Mrp2, Mrp4, Osta, CYP3A11 ↑; Butyrate production ↑; regulating CYP7A1, CYP8B1 ↑	Alleviates inflammation; Reduces toxic bile acid accumulation	(Fang et al., 2022; Wang et al., 2024a)
	Artemisia capillaris Thunb. Polysaccharide	Proliferation of SCFA-producing bacteria ↑	Butyrate production ↑; Hepatic Nrf2 signaling pathway ↑	Ameliorates cholestasis; Treats CLI ↑	(Cai et al., 2024)
	Salidroside	*Lactobacillus ↑;* *Bifidobacterium, Enterococcus ↓*	OCLN, ZO-1 ↑; PI3K/AKT/GSK-3β pathway ↑; Modulates SOD, GSH-Px, MDA, ALT, AST, TBIL, ALB; Regulates Bax/Bcl-2, caspase	Repairs intestinal barrier, reduces translocation of toxic substances	(Wang et al., 2024b)
	Baicalin	Proliferation of SCFA-producing bacteria ↑	Modulates TGR5, FXR; TLR4/NF-κB pathway ↓; Regulates Nrf2/Keap-1/HO-1 pathway; Caspase-3, Bax ↓	Reduces cholestasis; Inhibits hepatocyte fibrosis and apoptosis	(Hu et al., 2021)
	Berberine	Converted by Bacteroides and Bifidobacterium into dhBBR; *Bacteroidetes ↑, Firmicutes ↓*	TPH1, 5-HT ↓; cAMP/PKA, MAPK, STAT3 pathways ↓; CYP7A1, SHP ↑; ZO-1, mucin-2 ↑; NF-κB, MAPK ↓	Reduces oxidative stress and apoptosis; Alleviates bile acid accumulation and inflammatory response	(Tu et al., 2025; Wang et al., 2024c)
Active Ingredients of Chinese Medicinal Herbs	Paeoniae Radix Rubra Extract	*Coprococcus, Lactobacillus, Ruminococcus, Oscillospira ↑;* *Bacteroides, Escherichia ↓*	Secondary bile acids ↓; ALT, AST, ALP, GGT, TBIL, DBIL, TBA ↓	Alleviates hepatocyte edema and neutrophil infiltration	(Xu et al., 2022)
	Desmodium styracifolium (Osb.) Merr. Extract	*Parvibacter↑;* *Paenalcaligenes↓*	FXR, Bsep, CYP7A1, NTCP ↑;TBA ↓; ALT, AST, ALP, TBIL, DBIL ↓	Reduce liver cell necrosis and jaundice	(Zhang et al., 2025b)
TCM patent drugs	Wuzhi tablet	*Actinobacteria, Firmicutes ↑;* *Bacteroidetes, Proteobacteria, Verrucomicrobia ↓*	PXR pathway ↑; Modulates CYP7A1, CYP3A11; ALT, AST, ALP, TBIL ↓	Reduce liver damage, liver cell necrosis and jaundice	(Li et al., 2020)
	Pien-Tze-Huang	*Lactobacillaceae, Lactobacillus ↑*	CYP7A1, CYP8B1, CYP27A1, CYP3A11, SULT2A8 ↑; T-β-MCA, TCDCA, CA ↓; ALT, AST, ALP, TBIL, TBA ↓	Alleviate hepatic necrosis and inflammatory cell infiltration	(Cao et al., 2025)
	Yinzhihuang Formula	*Clostridiale ↑;* *Escherichia-Shigella ↓*	DCA, LCA, TCA, T-β-MCA ↓; CYP7A1, CYP7B1, CYP27A1 ↓; IL-6, TNF-α, ALT, ALP ↓	Reduce liver necrosis, inflammation and fibrosis	(Luo et al., 2025)
	Chidan Tuihuang Granule	*Gram-negative bacteria* ↓	DAP ↓; ZO-1, OCLN ↑; NOD1/RIPK2/NF-κB pathway ↓; TNF-α, IL-6, IL-1β, IL-18 ↓; caspase-3, caspase-9 ↓	Restore the intestinal barrier, alleviate liver inflammation and reduce liver cell apoptosis	(Chen et al., 2024)
Chinese herbal compound formula	Yinchenhao Decoction	*Bacteroides ↓, Akkermansia ↑;* *Roseburia ↑, Escherichia-Shigella ↓*	TBA, ALT, AST ↓; CDCA, DCA, CA ↑, FXR-FGF15 axis ↑, CYP7A1 ↓, Bsep, Mrp2 ↑; OCLN, Claudin-1 ↑	Reduce bile stasis and inflammation	(Zhao et al., 2024; Li et al., 2025b)
	Bie Jia Jian Pill	Modulate the composition and diversity of the gut microbiota	TMA→TMAO ↓; PI3K/AKT pathway ↓	Relieve liver pathological damage, inflammation and fibrosis	(Cui et al., 2025b)
	Si-Ni-San	*P. goldsteinii ↑*	secondary bile acids and tryptophan derivatives	Reduce inflammation in the intestines and liver	(Li et al., 2025a)
	Da-Huang-Xiao-Shi Decoction	*Lactobacillus ↑*	SCFAs ↑; CLDN1, OCLN, ZO-1 ↑; Modulates NTCP, CYP7A1, FXR, Mrp2, Bsep; TNF-α, IL-6, IL-1β, IL-10 ↓	Enhance the intestinal barrier function	(Wang et al., 2022b)
	Huangqi Decoction	*Prevotellaceae_NK3B31_group*, *Alistipes*, *Gordonibacter*↓	NLRP3↓	Reduce the deposition of collagen around the bile ducts	(Zou et al., 2021)

↑, upregulated; ↓, dwonregulated.

### 4.1 Single herbal extracts

#### 4.1.1 Gardenia jasminoides Ellis polysaccharide

In a mouse model of CLI, daily administration of GPS (50–400 mg/kg) (Fang et al., 2022) has been shown to increase the abundance of beneficial bacteria such as *Bacteroidetes* and *Lachnospiraceae*, while reducing the abundance of pathogenic bacteria including *Proteobacteria*, *Enterobacteriaceae*, and *Enterococcaceae*. It concurrently upregulates the expression of ZO-1 and OCLN, thereby reducing the translocation of lipopolysaccharide to the liver. This reduction inhibits the TLR4/NF-κB signaling pathway, leading to decreased expression of Monocyte Chemoattractant Protein-1 and IL-6. Furthermore, GPS upregulates the expression of FXR and the Pregnane X Receptor (PXR), along with their downstream targets including the bile salt export pump (Bsep), multidrug resistance-associated protein 2 (Mrp2), multidrug resistance-associated protein 4 (Mrp4), organic solute transporter alpha/beta, and the metabolic enzyme Cytochrome P450 Family 3 Subfamily A Polypeptide 11 (CYP3A11). These actions promote bile acid excretion and metabolism, restore bile acid homeostasis, and consequently alleviate CLI. In addition, it can promote the production of butyric acid by the gut microbiota, which subsequently activates FXR and inhibits the expression of key synthetic enzymes, including CYP7A1 and CYP8B1. This leads to a reduction in the accumulation of toxic bile acids such as taurochenodeoxycholic acid (TCDCA), taurocholic acid (TCA), and glycocholic acid (GCA). Furthermore, by inhibiting the TLR4/NF-κB signaling pathway, it decreases the expression of inflammatory cytokines including IL-1β, TNF-α, and IL-6, thereby mitigating immune-inflammatory responses and exerting protective effects against CLI, Furthermore, no apparent signs of toxicity were observed in normal control mice at a GPS dose of 200 mg/kg/day (Wang et al., 2024a).

However, geniposide, another component derived from Gardenia jasminoides, induces hepatotoxicity at a high dose (450 mg/kg) by disrupting bile acid homeostasis and activating the NLRP3 inflammasome (Jin et al., 2025). This underscores that the hepatic effects of substances derived from Gardenia jasminoides must be evaluated with careful consideration of their specific chemical constituents and administered dosage.

#### 4.1.2 Artemisia capillaris Thunb. polysaccharide

Studies have shown that administration of APS at 50 or 100 mg/kg/day for 7 consecutive days in mice ameliorates cholestasis and alleviates CLI by modulating the gut microbiota, specifically by increasing the abundance of SCFA-producing bacteria, thereby enhancing the production of butyrate. The elevated butyrate levels subsequently activate the hepatic Nrf2 signaling pathway (Cai et al., 2024).

#### 4.1.3 Salidroside

Treatment of CLI model mice with Salidroside (SAL) (5–20 mg/kg) for two weeks (Wang et al., 2024b) increases the abundance of beneficial bacteria such as *Lactobacillus* while reducing the abundance of harmful bacteria including *Bifidobacterium* and *Enterococcus*. It upregulates the expression of OCLN and ZO-1, thereby enhancing the intestinal barrier function. This action reduces the translocation of toxic substances from the gut to the liver, leading to decreased levels of serum and hepatic TBA as well as pro-inflammatory cytokines (TNF-α, IL-1β, and IL-6), which alleviates hepatic inflammation. Furthermore, SAL elevates the activities of SOD and glutathione peroxidase (GSH-Px), reduces malondialdehyde (MDA), and improves markers of liver function including alanine aminotransferase (ALT), aspartate aminotransferase (AST), total bilirubin (TBIL), and albumin (ALB). Additionally, it activates the PI3K/AKT/GSK-3β signaling pathway, resulting in a reduced Bax/Bcl-2 ratio and inhibited caspase cleavage. These effects collectively suppress hepatic stellate cell activation, proliferation, and migration, thereby ameliorating cholestatic liver fibrosis.

#### 4.1.4 Baicalin

Administration of baicalin (Hu et al., 2021) (50–200 mg/kg) by gavage for 5 consecutive days to CLI model rats can alleviate cholestasis primarily by promoting the production of SCFAs and modulating bile acid receptors such as TGR5 and FXR. This action subsequently blocks the TLR4/NF-κB signaling pathway, leading to a significant reduction in the levels of key inflammatory cytokines, including TNF-α, IL-1β, and IL-6. Furthermore, baicalin combats oxidative stress by regulating the Nrf2/Keap-1/HO-1 signaling pathway. It also downregulates apoptosis-related proteins like Caspase-3 and Bax, thereby inhibiting the fibrotic progression of cholestatic hepatocytes.

#### 4.1.5 Berberine

Research has found (Tu et al., 2025) that Oral administration of BBR (20 mg/kg/day) for 7 consecutive days markedly alleviated critical limb ischemia in a mouse model of CLI. Nitroreductase-producing bacteria, such as *Bacteroides* and *Bifidobacterium*, can convert BBR into its active metabolite dihydroberberine (dhBBR). Locally in the gut, dhBBR inhibits both the transcription and activity of tryptophan hydroxylase 1 (TPH1), thereby reducing the synthesis of 5-hydroxytryptamine (5-HT) in enterochromaffin cells. This action suppresses the activation of downstream signaling pathways, including cAMP/PKA, MAPK, and STAT3. Consequently, it improves liver injury markers in serum, such as ALT, AST, ALP, and TBIL, alleviates oxidative stress, mitochondrial dysfunction, and caspase-3-mediated apoptosis within hepatocytes, ultimately ameliorating hepatocyte necrosis. Another study found that BBR can alleviate CLI by increasing the relative abundance of *Bacteroidetes* and reducing the proportion of *Firmicutes*, which exhibit high bile salt hydrolase activity, in the mouse gut. Furthermore, BBR upregulates hepatic nuclear receptor genes involved in bile acid synthesis, such as the rate-limiting enzyme CYP7A1 and SHP, thereby reducing the accumulation of bile acids in the liver and subsequently mitigating endoplasmic reticulum stress and inflammatory responses. Concurrently, BBR enhances the expression of ZO-1 and mucin-2, reduces bacterial translocation, and ultimately inhibits the expression of inflammatory genes like IL-1β by downregulating the NF-κB and MAPK signaling pathways (Wang et al., 2024c).

### 4.2 Active ingredients of Chinese medicinal herbs

#### 4.2.1 Paeoniae radix rubra extract

In a rat model of CLI, treatment with PRR extract at a dose of 20 g/kg (equivalent to 55.48 g/kg of the crude drug) administered twice daily for 4 days (Xu et al., 2022) was found to upregulate the relative abundance of beneficial bacterial genera such as *Coprococcus*, *Lactobacillus*, *Ruminococcus*, and *Oscillospira*, while downregulating potentially harmful genera including *Bacteroides* and *Escherichia*, which are positively correlated with secondary bile acid levels. It also reduces secondary bile acids and serum levels of ALT, AST, ALP, GGT, TBIL, TBA, and direct bilirubin (DBIL). Furthermore, PRR alleviates hepatocyte edema and neutrophil infiltration, thereby mitigating CLI in rats.

#### 4.2.2 Desmodium styracifolium (Osb.) Merr. extract

Administering DME via gavage to a mouse model of CLI at doses ranging from 50 to 200 mg/kg for 11 consecutive days (Zhang et al., 2025b) ameliorates CLI by modulating the gut microbiota composition, notably increasing the abundance of *Parvibacter* and decreasing the abundance of *Paenalcaligenes*, and activating the FXR pathway. Specifically, it significantly upregulates the mRNA and protein expression of hepatic FXR, Bsep, CYP7A1, and Na^+^-taurocholate cotransporting polypeptide (NTCP). This leads to improved bile acid synthesis and reduced TBA levels in both serum and liver. DME lowers serum biochemical indicators—including ALT, AST, ALP, TBIL, DBIL, and TBA—in mice, while alleviating histopathological manifestations of cholestasis such as liver injury, necrosis, and jaundice.

### 4.3 TCM patent drugs

#### 4.3.1 Wuzhi tablet

In a study using an LCA-induced cholestatic mouse model, at a specific dose (350 mg/kg, administered orally for 7 days), WZ (Li et al., 2020) has been shown to increase the abundance of *Actinobacteria* and *Firmicutes* phyla while reducing the abnormal elevation of *Bacteroidetes*, *Proteobacteria*, and *Verrucomicrobia*. It activates the PXR pathway and modulates the expression of CYP7A1 and CYP3A11 genes, thereby promoting the hepatic excretion of toxic bile acids such as cholic acid (CA), TCA and TDCA. This reduces intrahepatic accumulation of bile acids and decreases serum levels of ALT, AST, ALP, and TBIL, suggesting its hepatoprotective effects under experimental conditions.

#### 4.3.2 Pien-Tze-Huang

In the LCA-induced cholestatic mouse model, consecutive 4-day administration of PTH (75–300 mg/kg) has been demonstrated to increase the abundance of *Lactobacillaceae* and *Lactobacillus*. It promotes the deconjugation of conjugated bile acids and upregulates the expression of key bile acid-related enzymes, including CYP7A1, CYP8B1, CYP27A1, CYP3A11, and SULT2A8. This leads to a reduction in excessively accumulated bile acids such as T-β-MCA, TCDCA, and TCA. Concurrently, PTH decreases serum levels of ALT, AST, ALP, TBIL, and TBA, alleviates hepatic necrosis and inflammatory cell infiltration, thereby mitigating LCA-induced CLI (Cao et al., 2025).

#### 4.3.3 Yinzhihuang formula

YZH (1.35, 2.7 and 5.4 g/kg, intragastric administration for 14 days, with 2.7 and 5.4 g/kg showing significant effects) (Luo et al., 2025) upregulates the abundance of probiotics associated with bile acid metabolism, such as *Clostridiale*, while downregulating pathogenic bacteria including *Escherichia-Shigella*. It promotes bile acid excretion, thereby reducing serum levels of hydrophobic bile acids like deoxycholic acid and lithocholic acid, as well as conjugated bile acids such as taurocholic acid and tauro-β-muricholic acid. Concurrently, YZH enhances bile salt hydrolase activity and suppresses the hepatic gene expression of key bile acid synthesis enzymes, namely CYP7A1, CYP7B1, and CYP27A1. Furthermore, YZH increases goblet cell numbers and tight junction protein expression, which helps restore intestinal barrier integrity and reduces bacterial translocation to the liver. These effects collectively lead to decreased hepatic mRNA expression of IL-6 and TNF-α, improved serum ALT and ALP levels, and amelioration of cholestatic hepatocellular necrosis, inflammatory infiltration, and fibrosis.

#### 4.3.4 Chidan Tuihuang granule

CDTH (Chen et al., 2024), at the dosages of 0.5, 1.5 and 3.0 g/kg with continuous intragastric administration for 7 days in rats, reduces the relative abundance of *Gram-negative bacteria*, thereby decreasing the production of their metabolic product, meso-2,6-diaminopimelic acid (DAP). Concurrently, CDTH upregulates the expression of ZO-1 and OCLN proteins, improving intestinal barrier function and inhibiting the translocation of DAP from the gut to the liver. This action downregulates the DAP-mediated NOD1/RIPK2 signaling pathway and suppresses the phosphorylation of NF-κB. Consequently, the expression of pro-inflammatory cytokines, including TNF-α, IL-6, IL-1β, and IL-18, is reduced, leading to attenuated hepatic inflammatory responses. Furthermore, CDTH diminishes hepatocyte apoptosis by decreasing the activity of caspase-3 and caspase-9. Collectively, these findings indicate that CDTH initiates its effects at the gut microbiota level and modulates immune-inflammatory pathways via the gut-liver axis, ultimately achieving hepatoprotective outcomes.

### 4.4 Chinese herbal compound formula

#### 4.4.1 Yinchenhao decoction

YCHD at a single gavage dose of 10 g/kg body weight (Zhao et al., 2024) reduces the abundance of pathogenic bacteria such as *Bacteroides* while increasing the abundance of beneficial bacteria such as *Akkermansia*. It lowers hepatic TBA and serum levels of ALT and AST, thereby ameliorating intrahepatic cholestasis and inflammatory injury. This establishes a regulatory axis along the gut microbiota–bile acid–liver axis. Further studies with consecutive gavage administration of YCHD at 3 g/kg and 9 g/kg body weight for 7 days (Li et al., 2025b) have shown that YCHD upregulates beneficial genera such as *Roseburia* and suppresses pathogenic genera such as *Escherichia-Shigella*, promoting the generation of CDCA, DCA, and CA. This activates the intestinal FXR-FGF15 signaling axis, which subsequently inhibits hepatic bile acid synthesis mediated by CYP7A1 and enhances the expression of hepatobiliary transporters such as Bsep and Mrp2, thereby restoring bile acid homeostasis. Moreover, YCHD upregulates the expression of tight junction proteins, including OCLN and claudin-1, which helps repair the intestinal barrier, reduce endotoxin translocation, and lower levels of ALT, AST, TBA, and TBIL. These effects collectively alleviate intestinal and hepatic inflammatory infiltration and mitigate CLI.

#### 4.4.2 Bie Jia Jian pill

BJJP (Cui et al., 2025b) was intragastrically administered to bile duct ligation (BDL) model rats at a gradient dose of 0.55 to 2.2 g/kg once daily for 14 days (an equivalent dose converted from clinical human usage with no obvious organ toxicity). It modulates the composition and diversity of the gut microbiota, thereby inhibiting the conversion of the microbial metabolite trimethylamine (TMA) to trimethylamine N-oxide (TMAO). This action suppresses the TMAO-mediated activation of the PI3K/AKT signaling pathway, consequently inhibiting the activation of hepatic stellate cells. Ultimately, BJJP ameliorates liver pathological damage, inflammation, and fibrosis in BDL model rats.

#### 4.4.3 Si-Ni-San

Gut microbiota depletion experiments and fecal microbiota transplantation (FMT) confirmed that the therapeutic benefits of SNS at the doses of 3.12 g/kg and 6.24 g/kg are dependent on gut microbiota modulation, particularly through the promotion of the growth of the potential probiotic bacterium *P. goldsteinii.* SNS ameliorates dysbiosis and modulates the profile of microbial metabolites, including beneficial secondary bile acids and tryptophan derivatives. These changes contribute to the alleviation of intestinal inflammation and restoration of intestinal barrier integrity, thereby mitigating liver inflammation and injury via the gut-liver axis (Li et al., 2025a).

#### 4.4.4 Da-Huang-Xiao-Shi decoction

In the α-naphthylisothiocyanate (ANIT)-induced cholestasis model of Sprague-Dawley rats, the experimentally effective dose of DHXSD (4.72 g/kg via intragastric administration for 7 consecutive days) (Wang et al., 2022b) upregulates the abundance of beneficial bacteria such as *Lactobacillus*, thereby promoting the production of SCFAs. This enhancement subsequently increases the expression of tight junction proteins, including claudin-1, OCLN, and ZO-1, leading to improved intestinal barrier function. DHXSD modulates the protein expression of NTCP, CYP7A1, FXR, MRP2, and Bsep. It reduces the levels of TNF-α, IL-6, and IL-1β while elevating the anti-inflammatory cytokine IL-10 in both the liver and intestine. It is worth noting that although DHXSD contains medicinal herbs such as Gardenia jasminoides and Rheum palmatum, and certain components like geniposide (Liu et al., 2025) and emodin (Zhang et al., 2020) may exhibit potential hepatotoxicity at high doses or with prolonged use, under the specific doses (Wang et al., 2022b) employed in this study, DHXSD as a whole demonstrated clear hepatoprotective effects rather than causing liver injury. Collectively, these actions ameliorate bile acid metabolic disorders and alleviate liver injury.

#### 4.4.5 Huangqi decoction

HQD at doses of 2 g/kg (equivalent to the human clinical dose) and 4 g/kg was intragastrically administered to mice once daily for 8 weeks (Zou et al., 2021). This treatment reduces the abundance of bacteria such as *Prevotellaceae_NK3B31_group, Alistipes*, and *Gordonibacter*, thereby alleviating gut barrier dysfunction caused by dysbiosis. Concurrently, it inhibits the activation of the NLRP3 inflammasome in the liver. These effects collectively attenuate hepatocyte injury and periductal collagen deposition, ultimately exerting a protective effect against CLI.

### 4.5 Comparative mechanistic delineation of TCM interventions on core signaling pathways in CLI

**Figure 4:**
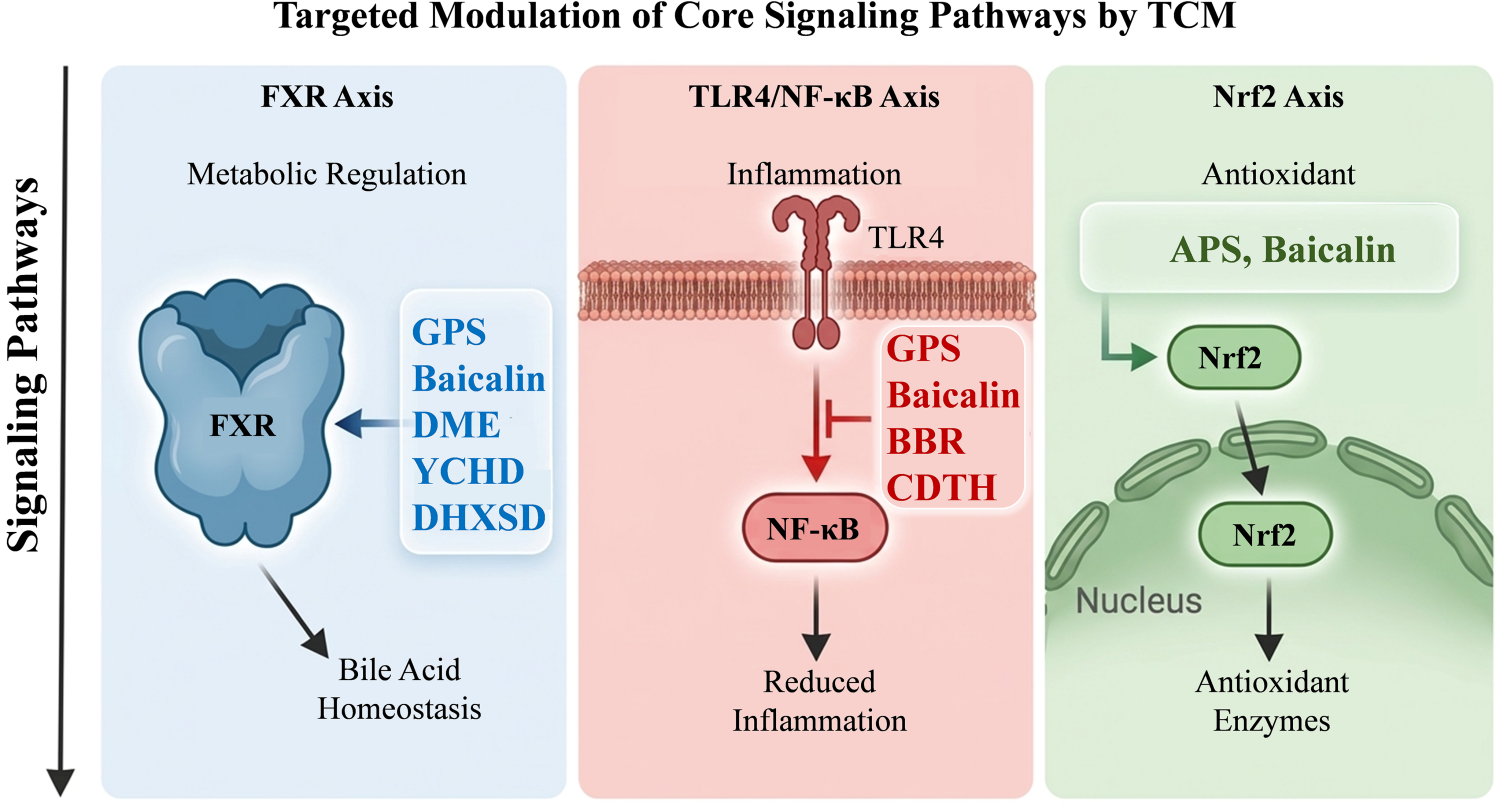
Comparative mechanistic framework of TCM interventions targeting core signaling pathways.

All TCM interventions evaluated in this review exert anti-cholestatic and hepatoprotective effects via gut microbiota modulation as the common upstream initiator, with their therapeutic actions functionally mapped to three core, cross-talking signaling pathways that target the cardinal pathological hallmarks of CLI (**Figure 4**).

The FXR signaling axis, the master regulator of enterohepatic bile acid homeostasis, is targeted by single herbal agents GPS (Wang et al., 2024a), baicalin (Hu et al., 2021) and DME (Zhang et al., 2025b), as well as classical compound formulas YCHD (Li et al., 2025b) and DHXSD (Wang et al., 2022b). These interventions reshape the gut microbiota to restore FXR signaling along the gut-liver axis, regulate bile acid synthesis and efflux, and mitigate hepatic accumulation of cytotoxic hydrophobic bile acids.

The TLR4/NF-κB signaling axis, the central driver of gut-liver inflammatory injury in CLI, is modulated by single herbal agents GPS (Wang et al., 2024a), baicalin (Hu et al., 2021) and BBR (Wang et al., 2024c), alongside the proprietary TCM preparation CDTH (Chen et al., 2024). These agents remodel the gut microbiota to repair intestinal barrier integrity, limit portal translocation of gut-derived pathogenic molecules, block TLR4/NF-κB-mediated inflammatory cascade activation, and attenuate hepatic inflammation and fibrosis.

The Nrf2 signaling axis, pivotal regulator of endogenous antioxidant defense, is targeted by single herbal extracts. APS (Cai et al., 2024) and baicalin (Hu et al., 2021) enrich SCFA-producing commensals via gut microbiota modulation, activate the Nrf2 cascade to enhance cellular redox homeostasis, and alleviate bile acid-induced oxidative hepatocellular damage.

Notably, these three pathways exhibit extensive bidirectional crosstalk, and most TCM agents simultaneously modulate multiple pathways, embodying the holistic, multi-target therapeutic advantages of TCM. This mechanistic mapping clarifies the distinct and complementary pharmacological profiles of TCM interventions, providing a rational basis for developing synergistic combinations and precision therapeutic strategies for CLI.

## 5 Conclusion and perspectives

CLI, as a complex liver disorder, is closely associated with intestinal flora dysregulation. This review systematically elaborates on the central role of the gut microbiota in CLI via the gut-liver axis, encompassing mechanisms such as the regulation of bile acid metabolism, mediation of immune-inflammatory responses, induction of oxidative stress, and participation in various signaling pathways. Furthermore, we have specifically highlighted the research progress in TCM regarding the modulation of the gut microbiota to ameliorate CLI. Accumulating evidence demonstrates that single herbal compounds, active ingredients, Chinese patent medicine, and Chinese herbal compound formulas can multi-targetedly and holistically restore bile acid homeostasis, alleviate liver inflammation and fibrosis, and exert therapeutic effects by reshaping the gut microbial structure, promoting the proliferation of beneficial bacteria, repairing intestinal barrier function, and regulating bile acid receptors and related signaling pathways.

Although current research has preliminarily revealed the potential of TCM in treating this disease through microbiota modulation, several issues warrant further in-depth exploration, as outlined below: a. Most studies remain at the preclinical stage, lacking validation from large-scale, multi-center, and well-designed clinical trials​ to confirm efficacy, safety, and reproducibility in human patients. b. The complex composition of Chinese herbal medicines makes the interaction mechanisms between their specific active components and the gut microbiota unclear. The specific pathways by which the microbiota metabolically transforms these components and their subsequent biological effects require further elucidation. Moreover, standardization remains a key challenge in the clinical translation of TCM for CLI treatment, due to the variability in herbal composition, cultivation conditions, and processing methods, which also affects the stability of TCM’s regulatory effects on gut microbiota. c. Significant inter-individual variability in gut microbiota poses a major challenge for achieving precision and personalized TCM therapies in the future. This variability may further influence the therapeutic responses to TCM interventions, making it necessary to incorporate microbiome profiling into clinical research to address individual differences.

Based on the aforementioned issues, future research directions are proposed as follows: a. Utilizing multi-omics approaches, such as metagenomics and metabolomics, as well as advanced analytical techniques including chromatography and mass spectrometry, to systematically elucidate the interaction network among TCM, the gut microbiota, and the host. These techniques can be used for accurate identification and quantification of bioactive compounds in TCM, laying a foundation for standardization. b. Conducting high-quality clinical research to clearly define the clinical efficacy of TCM in treating CLI via microbiota modulation. Meanwhile, establishing validated extraction protocols and quality control standards for TCM to ensure dose consistency, and incorporating microbiome profiling and personalized medicine approaches into clinical trials to better understand patient-specific responses, thereby ensuring reproducibility, safety, and efficacy in clinical applications. c. Strengthening research on the identification of active TCM components and their pharmacokinetics under the influence of the gut microbiota, thereby providing a theoretical basis for the development of new TCM-based drugs.

In conclusion, by focusing on the gut-liver axis and delving into the holistic regulatory advantages of TCM, new avenues are anticipated for the prevention and treatment of CLI.

## References

[B1] Akkız H. Gieseler R. K. Canbay A. (2024). Liver fibrosis: From basic science towards clinical progress, focusing on the central role of hepatic stellate cells. Int. J. Mol. Sci. 25, 7873. doi: 10.3390/ijms25147873 39063116 PMC11277292

[B2] Alam S. Lal B. B. (2022). Recent updates on progressive familial intrahepatic cholestasis types 1, 2 and 3: Outcome and therapeutic strategies. World J. Hepatol. 14, 98–118. doi: 10.4254/wjh.v14.i1.98 35126842 PMC8790387

[B3] Albillos A. De Gottardi A. Rescigno M. (2020). The gut-liver axis in liver disease: Pathophysiological basis for therapy. J. Hepatol. 72, 558–577. doi: 10.1016/j.jhep.2019.10.003 31622696

[B4] Ali A. I. El-Tawil O. S. El-Rahman S. S. A. (2021). Inhibited TLR-4/NF-κB pathway mediated by cannabinoid receptor 2 activation curbs ongoing liver fibrosis in bile duct ligated rats. Adv. Anim. Vet. 9, 253-264. doi: 10.17582/journal.aavs/2021/9.2.253.264, PMID: 41952288

[B5] Benedé-Ubieto R. Cubero F. J. Nevzorova Y. A. (2024). Breaking the barriers: the role of gut homeostasis in Metabolic-Associated Steatotic Liver Disease (MASLD). Gut Microbes 16, 2331460. doi: 10.1080/19490976.2024.2331460 38512763 PMC10962615

[B6] Bozward A. G. Ronca V. Osei-Bordom D. Oo Y. H. (2021). Gut-liver immune traffic: Deciphering immune-pathogenesis to underpin translational therapy. Front. Immunol. 12. doi: 10.3389/fimmu.2021.711217 34512631 PMC8425300

[B7] Bradley E. Haran J. (2024). The human gut microbiome and aging. Gut Microbes 16, 2359677. doi: 10.1080/19490976.2024.2359677 38831607 PMC11152108

[B8] Cai J. Zhu Z. Li Y. Li Q. Tian T. Meng Q. . (2024). Artemisia capillaris Thunb. Polysaccharide alleviates cholestatic liver injury through gut microbiota modulation and Nrf2 signaling pathway activation in mice. J. Ethnopharmacol. 327, 118009. doi: 10.1016/j.jep.2024.118009 38447617

[B9] Cao H. Chen L. Zeng Z. Wu X. Lei Y. Jia W. . (2024). Reversal of cholestatic liver disease by the inhibition of sphingosine 1-phosphate receptor 2 signaling. PeerJ 12, e16744. doi: 10.7717/peerj.16744 38250717 PMC10798156

[B10] Cao Y. Zhai Y. Deng Q. Song S. Li W. Li Y. . (2025). Pien-Tze-Huang alleviates lithocholic acid-induced cholestasis in mice by shaping bile acid-submetabolome. Chin. Med. 20, 103. doi: 10.1186/s13020-025-01161-7 40605096 PMC12225082

[B11] Casado-Bedmar M. Roy M. Berthet L. Hugot J. P. Yang C. Manceau H. . (2024). Fecal let-7b and miR-21 directly modulate the intestinal microbiota, driving chronic inflammation. Gut Microbes 16, 2394249. doi: 10.1080/19490976.2024.2394249 39224018 PMC11376420

[B12] Chang H. Jiang Y. Zhao Q. Su Z. Chen M. He Q. . (2025). Disruption of bile acid metabolism in the gut-liver axis predisposes mice to inflammatory bowel disease. MedComm (2020) 6, e70429. doi: 10.1002/mco2.70429 41079644 PMC12508617

[B13] Chen W. Wei Y. Xiong A. Li Y. Guan H. Wang Q. . (2020). Comprehensive analysis of serum and fecal bile acid profiles and interaction with gut microbiota in primary biliary cholangitis. Clin. Rev. Allergy Immunol. 58, 25–38. doi: 10.1007/s12016-019-08731-2 30900136

[B14] Chen Y. Hu Q. Zhang W. Gong Q. Yan J. Wang Z. . (2024). Chidan Tuihuang granule modulates gut microbiota to influence NOD1/RIPK2 pathway in cholestatic liver injury recovery. Phytomedicine 135, 156164. doi: 10.1016/j.phymed.2024.156164 39461197

[B15] Collins S. L. Stine J. G. Bisanz J. E. Okafor C. D. Patterson A. D. (2023). Bile acids and the gut microbiota: metabolic interactions and impacts on disease. Nat. Rev. Microbiol. 21, 236–247. doi: 10.1038/s41579-022-00805-x 36253479 PMC12536349

[B16] Crovesy L. Masterson D. Rosado E. L. (2020). Profile of the gut microbiota of adults with obesity: a systematic review. Eur. J. Clin. Nutr. 74, 1251–1262. doi: 10.1038/s41430-020-0607-6 32231226

[B17] Cui C. Gao S. Shi J. Wang K. (2025a). Gut-liver axis: The role of intestinal microbiota and their metabolites in the progression of metabolic dysfunction-associated steatotic liver disease. Gut Liver 19, 479–507. doi: 10.5009/gnl240539 40336226 PMC12261135

[B18] Cui X. Zhang R. Li Y. Li P. Liu Y. Yu X. . (2025b). Bie Jia Jian pill ameliorates BDL-induced cholestatic hepatic fibrosis in rats by regulating intestinal microbial composition and TMAO-mediated PI3K/AKT signaling pathway. J. Ethnopharmacol. 337, 118910. doi: 10.1016/j.jep.2024.118910 39369915

[B19] Đurašević S. Pejić S. Grigorov I. Nikolić G. Mitić-Ćulafić D. Dragićević M. . (2021). Effects of C60 fullerene on thioacetamide-induced rat liver toxicity and gut microbiome changes. Antioxidants (Basel) 10, 911. doi: 10.3390/antiox10060911 34199786 PMC8226855

[B20] Fan S. X. Wang J. Li Q. Li Y. S. Guan W. X. Li J. S. (2021). Mechanism of gut-microbiota-liver axis in the pathogenesis of intestinal failure-associated liver disease. Zhonghua Wei Chang Wai Ke Za Zhi 24, 94–100. doi: 10.3760/cma.j.cn.441530-20201009-00550 33461259

[B21] Fang S. Wang T. Li Y. Xue H. Zou J. Cai J. . (2022). Gardenia jasminoides Ellis polysaccharide ameliorates cholestatic liver injury by alleviating gut microbiota dysbiosis and inhibiting the TLR4/NF-κB signaling pathway. Int. J. Biol. Macromol 205, 23–36. doi: 10.1016/j.ijbiomac.2022.02.056 35176320

[B22] Feng T. Yu H. Ye L. (2025). Mechanisms and strategies for engineering oxidative stress resistance in Saccharomyces cerevisiae. Chem. Bio Eng 2, 409–422. doi: 10.1021/cbe.5c00021 40735014 PMC12301939

[B23] Fusco W. Lorenzo M. B. Cintoni M. Porcari S. Rinninella E. Kaitsas F. . (2023). Short-chain fatty-acid-producing bacteria: Key components of the human gut microbiota. Nutrients 15, 2211. doi: 10.3390/nu15092211 37432351 PMC10180739

[B24] Garcia-Llorens G. El Ouardi M. Valls-Belles V. (2025). Oxidative stress fundamentals: Unraveling the pathophysiological role of redox imbalance in non-communicable diseases. Appl. Sci. 15, 10191. doi: 10.3390/app151810191 41725453

[B25] Grüner N. Mattner J. (2021). Bile acids and microbiota: Multifaceted and versatile regulators of the liver-gut axis. Int. J. Mol. Sci. 22, 1397. doi: 10.3390/ijms22031397 33573273 PMC7866539

[B26] Gulamhusein A. F. Hirschfield G. M. (2020). Primary biliary cholangitis: pathogenesis and therapeutic opportunities. Nat. Rev. Gastroenterol. Hepatol. 17, 93–110. doi: 10.1038/s41575-019-0226-7 31819247

[B27] Guo Z. He K. Pang K. Yang D. Lyu C. Xu H. . (2024). Exploring advanced therapies for primary biliary cholangitis: Insights from the gut microbiota-bile acid-immunity network. Int. J. Mol. Sci. 25, 4321. doi: 10.3390/ijms25084321 38673905 PMC11050225

[B28] Hahn L. Helmrich N. Herebian D. Mayatepek E. Drebber U. Domann E. . (2020). IL-13 as target to reduce cholestasis and dysbiosis in Abcb4 knockout mice. Cells 9, 1949. doi: 10.3390/cells9091949 32846954 PMC7564366

[B29] He S. L. Li Z. H. Li J. Li Y. (2025). The interaction between IL-17 and gut microbiota contributes to cholestatic liver disease in children. Microbiol. (Reading) 171, 001608. doi: 10.1099/mic.0.001608 40956601 PMC12453119

[B30] Hernandez G. V. Smith V. A. Melnyk M. Burd M. A. Sprayberry K. A. Edwards M. S. . (2020). Dysregulated FXR-FGF19 signaling and choline metabolism are associated with gut dysbiosis and hyperplasia in a novel pig model of pediatric NASH. Am. J. Physiol. Gastrointest Liver Physiol. 318, G582–g609. doi: 10.1152/ajpgi.00344.2019 32003601 PMC7099491

[B31] Hsu C. L. Schnabl B. (2023). The gut-liver axis and gut microbiota in health and liver disease. Nat. Rev. Microbiol. 21, 719–733. doi: 10.1038/s41579-023-00904-3 37316582 PMC10794111

[B32] Hu Q. Zhang W. Wu Z. Tian X. Xiang J. Li L. . (2021). Baicalin and the liver-gut system: Pharmacological bases explaining its therapeutic effects. Pharmacol. Res. 165, 105444. doi: 10.1016/j.phrs.2021.105444 33493657

[B33] Huang M. X. Yang S. Y. Luo P. Y. Long J. Liu Q. Z. Wang J. . (2021). Gut microbiota contributes to sexual dimorphism in murine autoimmune cholangitis. J. Leukoc. Biol. 110, 1121–1130. doi: 10.1002/jlb.3ma0321-037r 34047390

[B34] Huang C. Y. Zhang H. P. Han W. J. Zhao D. T. Liao H. Y. Ma Y. X. . (2022). Disease predisposition of human leukocyte antigen class II genes influences the gut microbiota composition in patients with primary biliary cholangitis. Front. Immunol. 13. doi: 10.3389/fimmu.2022.984697 36203614 PMC9531677

[B35] Isaacs-Ten A. Echeandia M. Moreno-Gonzalez M. Brion A. Goldson A. Philo M. . (2020). Intestinal microbiome-macrophage crosstalk contributes to cholestatic liver disease by promoting intestinal permeability in mice. Hepatology 72, 2090–2108. doi: 10.1002/hep.31228 32168395 PMC7839474

[B36] Jensen N. Maldonado-Gomez M. Krishnakumar N. Weng C. Y. Castillo J. Razi D. . (2024). Dietary fiber monosaccharide content alters gut microbiome composition and fermentation. Appl. Environ. Microbiol. 90, e0096424. doi: 10.1128/aem.00964-24 39007602 PMC11337808

[B37] Jiang H. Yu Y. Hu X. Du B. Shao Y. Wang F. . (2024). The fecal microbiota of patients with primary biliary cholangitis (PBC) causes PBC-like liver lesions in mice and exacerbates liver damage in a mouse model of PBC. Gut Microbes 16, 2383353. doi: 10.1080/19490976.2024.2383353 39105259 PMC11305030

[B38] Jiao N. Baker S. S. Chapa-Rodriguez A. Liu W. Nugent C. A. Tsompana M. . (2018). Suppressed hepatic bile acid signalling despite elevated production of primary and secondary bile acids in NAFLD. Gut 67, 1881–1891. doi: 10.1136/gutjnl-2017-314307 28774887

[B39] Jin Z. Cheng S. Liu B. Liu Y. Wei Y. Hao X. . (2025). Geniposide induces hepatotoxicity via the bile acid-induced activation of NLRP3 inflammasome and regulation of the FXR/PERK/TXNIP pathway. Toxicol. Lett. 413, 111740. doi: 10.1016/j.toxlet.2025.111740 41043658

[B40] Juanola O. Hassan M. Kumar P. Yilmaz B. Keller I. Simillion C. . (2021). Intestinal microbiota drives cholestasis-induced specific hepatic gene expression patterns. Gut Microbes 13, 1–20. doi: 10.1080/19490976.2021.1911534 33847205 PMC8049203

[B41] Kandalgaonkar M. R. Kumar V. Vijay-Kumar M. (2024). Digestive dynamics: Unveiling interplay between the gut microbiota and the liver in macronutrient metabolism and hepatic metabolic health. Physiol. Rep. 12, e16114. doi: 10.14814/phy2.16114 38886098 PMC11182692

[B42] Kennedy L. L. Meng F. Venter J. K. Zhou T. Karstens W. A. Hargrove L. A. . (2016). Knockout of microRNA-21 reduces biliary hyperplasia and liver fibrosis in cholestatic bile duct ligated mice. Lab. Invest 96, 1256–1267. doi: 10.1038/labinvest.2016.112 27775690 PMC5121007

[B43] Khalil M. Di Ciaula A. Mahdi L. Jaber N. Di Palo D. M. Graziani A. . (2024). Unraveling the role of the human gut microbiome in health and diseases. Microorganisms 12, 2333. doi: 10.3390/microorganisms12112333 39597722 PMC11596745

[B44] Kim S. Y. Chae C. W. Lee H. J. Jung Y. H. Choi G. E. Kim J. S. . (2020). Sodium butyrate inhibits high cholesterol-induced neuronal amyloidogenesis by modulating NRF2 stabilization-mediated ROS levels: involvement of NOX2 and SOD1. Cell Death Dis. 11, 469. doi: 10.1038/s41419-020-2663-1 32555166 PMC7303181

[B45] Larabi A. B. Masson H. L. P. Bäumler A. J. (2023). Bile acids as modulators of gut microbiota composition and function. Gut Microbes 15, 2172671. doi: 10.1080/19490976.2023.2172671 36740850 PMC9904317

[B46] Leung H. Xiong L. Ni Y. Busch A. Bauer M. Press A. T. . (2023). Impaired flux of bile acids from the liver to the gut reveals microbiome-immune interactions associated with liver damage. NPJ Biofilms Microbiomes 9, 35. doi: 10.1038/s41522-023-00398-0 37286586 PMC10247725

[B47] Li D. S. Huang Q. F. Guan L. H. Zhang H. Z. Li X. Fu K. L. . (2020). Targeted bile acids and gut microbiome profiles reveal the hepato-protective effect of WZ tablet (Schisandra sphenanthera extract) against LCA-induced cholestasis. Chin. J. Nat. Med. 18, 211–218. doi: 10.1016/s1875-5364(20)30023-6 32245591

[B48] Li D. Wan M. Xue L. Zhang Z. Qiu Y. Mei F. . (2024). Zinc promotes microbial p-coumaric acid production that protects against cholestatic liver injury. Cell Host Microbe 32, 2195–2211.e9. doi: 10.1016/j.chom.2024.11.002 39610253

[B49] Li F. Han Q. Cai Y. Li Y. Yang Y. Li J. . (2025a). Si-Ni-San ameliorates cholestatic liver injury by favoring P. goldsteinii colonization. J. Ethnopharmacol. 337, 118804. doi: 10.1016/j.jep.2024.118804 39270883

[B50] Li W. Huang D. Luo Z. Zhou T. Jin Z. (2025b). Yinchenhao decoction mitigates cholestatic liver injury in mice via gut microbiota regulation and activation of FXR-FGF15 pathway. Pharm. (Basel) 18, 932. doi: 10.3390/ph18070932 40732223 PMC12300480

[B51] Li X. Xie H. Chao J. J. Jia Y. H. Zuo J. An Y. P. . (2023). Profiles and integration of the gut microbiome and fecal metabolites in severe intrahepatic cholestasis of pregnancy. BMC Microbiol. 23, 282. doi: 10.1186/s12866-023-02983-x 37784030 PMC10546765

[B52] Liu X. Kang W. Li J. Li X. Yang P. Shi M. . (2024). Melatonin ameliorates cadmium-induced liver fibrosis via modulating gut microbiota and bile acid metabolism. J. Pineal Res. 76, e70005. doi: 10.1111/jpi.70005 39555739

[B53] Liu Y. Liu B. Shi M. Ye T. Li H. (2025). NLRP3 inflammasome activation is involved in geniposide-induced hepatotoxicity. Mediators Inflamm. 2025, 4112856. doi: 10.1155/mi/4112856 39949920 PMC11824841

[B54] Lu Y. Feng X. Wang Z. Zou M. Xu Z. Liu Q. . (2025). Bile acid metabolism and hcepatocellular carcinoma: mechanisms of drug resistance and intervention strategies. Precis Clin. Med. 8, pbaf020. doi: 10.1093/pcmedi/pbaf020 40980688 PMC12448427

[B55] Luo X. Cheng P. Fang Y. Wang F. Mao T. Shan Y. . (2025). Yinzhihuang formula modulates the microbe–gut–liver axis and bile acid excretion to attenuate cholestatic liver injury. Phytomedicine 139, 156495. doi: 10.1016/j.phymed.2025.156495 39978276

[B56] Luo X. You X. (2023). Genetic predisposition of the gastrointestinal microbiome and primary biliary cholangitis: a bi-directional, two-sample Mendelian randomization analysis. Front. Endocrinol. (Lausanne) 14. doi: 10.3389/fendo.2023.1225742 37900141 PMC10602727

[B57] Martin-Gallausiaux C. Marinelli L. Blottière H. M. Larraufie P. Lapaque N. (2021). SCFA: mechanisms and functional importance in the gut. Proc. Nutr. Soc 80, 37–49. doi: 10.1017/s0029665120006916 32238208

[B58] McCallum G. Tropini C. (2024). The gut microbiota and its biogeography. Nat. Rev. Microbiol. 22, 105–118. doi: 10.1038/s41579-023-00969-0 37740073

[B59] Mol B. Werner E. Culver E. L. van der Meer A. J. Bogaards J. A. Ponsioen C. Y. (2025). Epidemiological and economical burden of cholestatic liver disease. Hepatology 82, 813–833. doi: 10.1097/hep.0000000000001341 40168457

[B60] Nguyen J. T. Riessen R. Zhang T. Kieffer C. Anakk S. (2021). Deletion of intestinal SHP impairs short-term response to cholic acid challenge in male mice. Endocrinology 162, bqab063. doi: 10.1210/endocr/bqab063 33769482 PMC8256632

[B61] Nie Q. Luo X. Wang K. Ding Y. Jia S. Zhao Q. . (2024). Gut symbionts alleviate MASH through a secondary bile acid biosynthetic pathway. Cell 187, 2717–2734.e33. doi: 10.1016/j.cell.2024.03.034 38653239

[B62] Park J. W. Kim J. H. Kim S. E. Jung J. H. Jang M. K. Park S. H. . (2022). Primary biliary cholangitis and primary sclerosing cholangitis: current knowledge of pathogenesis and therapeutics. Biomedicines 10, 1288. doi: 10.3390/biomedicines10061288 35740310 PMC9220082

[B63] Paul J. K. Azmal M. Haque A. Meem M. Talukder O. F. Ghosh A. (2025). Unlocking the secrets of the human gut microbiota: comprehensive review on its role in different diseases. World J. Gastroenterol. 31, 99913. doi: 10.3748/wjg.v31.i5.99913 39926224 PMC11718612

[B64] Roberts S. B. Choi W. J. Worobetz L. Vincent C. Flemming J. A. Cheung A. . (2024). Loss of biochemical response at any time worsens outcomes in UDCA-treated patients with primary biliary cholangitis. JHEP Rep. 6, 101168. doi: 10.1016/j.jhepr.2024.101168 39380718 PMC11460452

[B65] Saeedi B. J. Liu K. H. Owens J. A. Hunter-Chang S. Camacho M. C. Eboka R. U. . (2020). Gut-resident lactobacilli activate hepatic Nrf2 and protect against oxidative liver injury. Cell Metab. 31, 956–968.e5. doi: 10.1016/j.cmet.2020.03.006 32213347 PMC7329068

[B66] Santos A. A. Afonso M. B. Ramiro R. S. Pires D. Pimentel M. Castro R. E. . (2020). Host miRNA-21 promotes liver dysfunction by targeting small intestinal lactobacillus in mice. Gut Microbes 12, 1–18. doi: 10.1080/19490976.2020.1840766 33300439 PMC7733982

[B67] Schnabl B. (2013). Linking intestinal homeostasis and liver disease. Curr. Opin. Gastroenterol. 29, 264–270. doi: 10.1097/MOG.0b013e32835ff948 23493073 PMC4077188

[B68] Shen X. Zhang X. Li K. Huang G. Li X. Hou Y. . (2024). Combined bacterial translocation and cholestasis aggravates liver injury by activation pyroptosis in obstructive jaundice. Heliyon 10, e35793. doi: 10.1016/j.heliyon.2024.e35793 39220957 PMC11363856

[B69] Stoeva M. K. Garcia-So J. Justice N. Myers J. Tyagi S. Nemchek M. . (2021). Butyrate-producing human gut symbiont, Clostridium butyricum, and its role in health and disease. Gut Microbes 13, 1–28. doi: 10.1080/19490976.2021.1907272 33874858 PMC8078720

[B70] Tan Z. Chen L. Ye Z. Lu Q. (2025). Xiaohuang Qudan decoction alleviates ANIT-induced cholestatic liver injury by inhibiting the JAK2/STAT3 pathway and regulating TH17/Treg. Chin. J. Nat. Med. 23, 457–470. doi: 10.1016/s1875-5364(25)60854-5 40274348

[B71] Tang X. Ning J. Zhao Y. Feng S. Shao L. Liu T. . (2025). Intestine-derived fibroblast growth factor 19 alleviates lipopolysaccharide-induced liver injury by regulating bile acid homeostasis and directly improving oxidative stress. J. Intensive Med. 5, 79–88. doi: 10.1016/j.jointm.2024.06.003 39872844 PMC11763227

[B72] Tilg H. Adolph T. E. Trauner M. (2022). Gut-liver axis: pathophysiological concepts and clinical implications. Cell Metab. 34, 1700–1718. doi: 10.1016/j.cmet.2022.09.017 36208625

[B73] Tokaç M. Aydin S. Taner G. Özkardeş A. B. Yavuz Taşlipinar M. Doğan M. . (2015). Hepatoprotective and antioxidant effects of lycopene in acute cholestasis. Turk J. Med. Sci. 45, 857–864. doi: 10.3906/sag-1404-57 26422858

[B74] Trauner M. Fuchs C. D. (2022). Novel therapeutic targets for cholestatic and fatty liver disease. Gut 71, 194–209. doi: 10.1136/gutjnl-2021-324305 34615727 PMC8666813

[B75] Trivedi P. J. Corpechot C. Pares A. Hirschfield G. M. (2016). Risk stratification in autoimmune cholestatic liver diseases: opportunities for clinicians and trialists. Hepatology 63, 644–659. doi: 10.1002/hep.28128 26290473 PMC4864755

[B76] Tu D. Lu C. Guo J. Chen Q. Li X. Wang Y. . (2025). Gut microbiota-mediated berberine metabolism ameliorates cholestatic liver disease by suppressing 5-HT production. Clin. Mol. Hepatol. 32, 221–238. doi: 10.3350/cmh.2025.0577 41087029 PMC12835802

[B77] Verkade E. Shen W. Hovingh M. V. Mulder N. L. de Bruyn K. Koehorst M. . (2023). Gut microbiota depletion aggravates bile acid-induced liver pathology in mice with a human-like bile acid composition. Clin. Sci. (Lond) 137, 1637–1650. doi: 10.1042/cs20230812 37910096 PMC10643054

[B78] Wang C. Zhao S. Xu Y. Sun W. Feng Y. Liang D. . (2022a). Integrated microbiome and metabolome analysis reveals correlations between gut microbiota components and metabolic profiles in mice with methotrexate-induced hepatoxicity. Drug Des. Devel Ther. 16, 3877–3891. doi: 10.2147/dddt.S381667 36388083 PMC9653027

[B79] Wang J. Wang X. Zhuo E. Chen B. Chan S. (2025a). Gut-liver axis in liver disease: from basic science to clinical treatment (review). Mol. Med. Rep. 31, 10. doi: 10.3892/mmr.2024.13375 39450549 PMC11541166

[B80] Wang J. Zhong M. Y. Liu Y. X. Yu J. Y. Wang Y. B. Zhang X. J. . (2025b). Branched-chain amino acids promote hepatic Cyp7a1 expression and bile acid synthesis via suppressing FGF21-ERK pathway. Acta Pharmacol. Sin. 46, 662–671. doi: 10.1038/s41401-024-01417-2 39567750 PMC11845675

[B81] Wang T. Tian T. Zhu Z. Fang S. Zhang L. Peng X. . (2024a). Gardenia jasminoides Ellis. polysaccharides alleviated cholestatic liver injury by increasing the production of butyric acid and FXR activation. Phytother Res. 38, 5363–5375. doi: 10.1002/ptr.8326 39237123

[B82] Wang Y. Zhao D. Su L. Tai Y. L. Way G. W. Zeng J. . (2024c). Therapeutic potential of berberine in attenuating cholestatic liver injury: insights from a PSC mouse model. Cell Biosci. 14, 14. doi: 10.1186/s13578-024-01195-8 38273376 PMC10809567

[B83] Wang X. Cao S. Huang Y. Li L. Xu D. Liu L. (2024b). Salidroside alleviates cholestasis-induced liver fibrosis by inhibiting hepatic stellate cells via activation of the PI3K/AKT/GSK-3β signaling pathway and regulating intestinal flora distribution. Front. Pharmacol. 15. doi: 10.3389/fphar.2024.1396023 38808258 PMC11130389

[B84] Wang W. Jiang S. Xu C. Tang L. Liang Y. Zhao Y. . (2022b). Transcriptome and gut microbiota profiling analysis of ANIT-induced cholestasis and the effects of Da-Huang-Xiao-Shi decoction intervention. Microbiol. Spectr 10, e0324222. doi: 10.1128/spectrum.03242-22 36409145 PMC9769994

[B85] Wei S. Wang R. Chen L. Jing M. Li H. Zheng R. . (2024). The contribution of small heterodimer partner to the occurrence and progression of cholestatic liver injury. J. Gastroenterol. Hepatol. 39, 1134–1144. doi: 10.1111/jgh.16544 38615196

[B86] Woolbright B. L. Jaeschke H. (2019). Measuring apoptosis and necrosis in cholestatic liver injury. Methods Mol. Biol. 1981, 133–147. doi: 10.1007/978-1-4939-9420-5_9 31016652

[B87] Wu L. Lv L. Xiang Y. Yi D. Liang Q. Ji M. . (2025). Rosmarinic acid protects against acetaminophen-induced hepatotoxicity by suppressing ferroptosis and oxidative stress through Nrf2/HO-1 activation in mice. Mar. Drugs 23, 287. doi: 10.3390/md23070287 40710512 PMC12299429

[B88] Wu L. Zhou J. Zhou A. Lei Y. Tang L. Hu S. . (2024). Lactobacillus acidophilus ameliorates cholestatic liver injury through inhibiting bile acid synthesis and promoting bile acid excretion. Gut Microbes 16, 2390176. doi: 10.1080/19490976.2024.2390176 39205654 PMC11364073

[B89] Xie X.-M. Zhang B.-Y. Feng S. Fan Z.-J. Wang G.-Y. (2025). Activation of gut FXR improves the metabolism of bile acids, intestinal barrier, and microbiota under cholestatic condition caused by GCDCA in mice. Microbiol. Spectr. 13, e03150-24. doi: 10.1128/spectrum.03150-24 39982108 PMC11960106

[B90] Xu J. J. Xu F. Wang W. Wang P. P. Xian J. Han X. . (2022). Paeoniae Radix Rubra can enhance fatty acid β-oxidation and alleviate gut microbiota disorder in α-naphthyl isothiocyanate induced cholestatic model rats. Front. Pharmacol. 13. doi: 10.3389/fphar.2022.1002922 36339580 PMC9633937

[B91] Yang S. Chang N. Li W. Yang T. Xue R. Liu J. . (2023b). Necroptosis of macrophage is a key pathological feature in biliary atresia via GDCA/S1PR2/ZBP1/p-MLKL axis. Cell Death Dis. 14, 175. doi: 10.1038/s41419-023-05615-4 36859525 PMC9977961

[B92] Yang H. Kuang Y. Wang L. Ma X. Gálvez J. A. V. Lu J. . (2025). Pterostilbene attenuates intestinal barrier damage and secondary liver oxidative stress in a murine model of Clostridium difficile infection by regulating the gut microbiota. Food Funct. 16, 3325–3343. doi: 10.1039/d4fo06413e 40190207 PMC12333533

[B93] Yang J. Y. Kweon M. N. (2016). The gut microbiota: a key regulator of metabolic diseases. BMB Rep. 49, 536–541. doi: 10.5483/bmbrep.2016.49.10.144 27530685 PMC5227294

[B94] Yang J. Tang X. Liang Z. Chen M. Sun L. (2023a). Taurocholic acid promotes hepatic stellate cell activation via S1PR2/p38 MAPK/YAP signaling under cholestatic conditions. Clin. Mol. Hepatol. 29, 465–481. doi: 10.3350/cmh.2022.0327 36800698 PMC10121313

[B95] Yang X. Xu Y. Li J. Ran X. Gu Z. Song L. . (2024). Bile acid-gut microbiota imbalance in cholestasis and its long-term effect in mice. mSystems 9, e0012724. doi: 10.1128/msystems.00127-24 38934542 PMC11265269

[B96] Zafar H. Saier M. H. (2021). Gut Bacteroides species in health and disease. Gut Microbes 13, 1–20. doi: 10.1080/19490976.2020.1848158 33535896 PMC7872030

[B97] Zeng H. Larson K. J. Cheng W. H. Bukowski M. R. Safratowich B. D. Liu Z. . (2020). Advanced liver steatosis accompanies an increase in hepatic inflammation, colonic, secondary bile acids and Lactobacillaceae/Lachnospiraceae bacteria in C57BL/6 mice fed a high-fat diet. J. Nutr. Biochem. 78, 108336. doi: 10.1016/j.jnutbio.2019.108336 32004929

[B98] Zhang Z. Guan G. Tang Z. Wan W. Huang Z. Wang Y. . (2025b). Desmodium styracifolium (Osb.) Merr. extracts alleviate cholestatic liver disease by FXR pathway. J. Ethnopharmacol. 337, 118972. doi: 10.1016/j.jep.2024.118972 39454708

[B99] Zhang P. Li X. Liang J. Zheng Y. Tong Y. Shen J. . (2025a). Chenodeoxycholic acid modulates cholestatic niche through FXR/Myc/P-selectin axis in liver endothelial cells. Nat. Commun. 16, 2093. doi: 10.1038/s41467-025-57351-2 40025016 PMC11873286

[B100] Zhang L. Yang L. Chu H. (2023). Targeting gut microbiota for the treatment of primary biliary cholangitis: from bench to bedside. J. Clin. Transl. Hepatol. 11, 958–966. doi: 10.14218/jcth.2022.00408 37408823 PMC10318297

[B101] Zhang Y. Yang X. Jia Z. Liu J. Yan X. Dai Y. . (2020). Proteomics unravels emodin causes liver oxidative damage elicited by mitochondrial dysfunction. Front. Pharmacol. 11. doi: 10.3389/fphar.2020.00416 32410985 PMC7201015

[B102] Zhao X. Wu X. Hu Q. Yao J. Yang Y. Wan M. . (2024). Yinchenhao decoction protects against acute liver injury in mice with biliary acute pancreatitis by regulating the gut microflora-bile acids-liver axis. Gastroenterol. Res. Pract. 2024, 8882667. doi: 10.1155/2024/8882667 38966598 PMC11223911

[B103] Zheng M. Zhai Y. Yu Y. Shen J. Chu S. Focaccia E. . (2024). TNF compromises intestinal bile-acid tolerance dictating colitis progression and limited infliximab response. Cell Metab. 36, 2086–2103.e9. doi: 10.1016/j.cmet.2024.06.008 38971153

[B104] Zhou T. Jin Z. Jiang R. Li W. (2025). Gut microbiota modulation by traditional Chinese medicine: a translational strategy for metabolic dysfunction-associated steatotic liver disease. Front. Pharmacol. 16. doi: 10.3389/fphar.2025.1600439 40556760 PMC12185430

[B105] Zhu F. Zheng S. Zhao M. Shi F. Zheng L. Wang H. (2023). The regulatory role of bile acid microbiota in the progression of liver cirrhosis. Front. Pharmacol. 14. doi: 10.3389/fphar.2023.1214685 37416060 PMC10320161

[B106] Zou J. Li W. Wang G. Fang S. Cai J. Wang T. . (2021). Hepatoprotective effects of Huangqi decoction (Astragali Radix and Glycyrrhizae Radix et Rhizoma) on cholestatic liver injury in mice: Involvement of alleviating intestinal microbiota dysbiosis. J. Ethnopharmacol. 267, 113544. doi: 10.1016/j.jep.2020.113544 33152436

[B107] Zou Y. Ni W. Zhou Y. Sun D. Chen F. Li X. (2025). Gut microbiota dysbiosis in infantile cholestatic hepatopathy. Front. Pediatr. 13. doi: 10.3389/fped.2025.1547958 40196165 PMC11973382

